# [Retracted] P21 activated kinase 2 promotes pancreatic cancer growth and metastasis

**DOI:** 10.3892/ol.2023.13949

**Published:** 2023-07-10

**Authors:** Guo-Wang Yao, Jing-Rui Bai, Da-Peng Zhang

Oncol Lett 17: 3709–3718, 2019; DOI: 10.3892/ol.2019.10040

Following the publication of this article and a corrigendum relating to its content (doi: 10.3892/ol.2020.11950) wherein a corrected version of Fig. 5 was presented, a concerned reader drew to the attention of the Editorial Office that the images selected for the western blot experiments in Fig. 5C were also included, albeit in different form, in the following paper written by different authors at different research institutes in the same city [Zhao C-C, Chen J, Niu R-F, Liu Y and Zhang C-G: Increased resistin suggests poor prognosis and promotes development of lung adenocarcinoma. Oncol Rep 40: 3392-3404, 2018]. Moreover, a range of data included in this paper also appeared in different formats in several other articles published subsequently.

Owing to the fact that the contentious data in Fig. 5 of the above article were already under consideration for publication prior to its submission to *Oncology Letters*, the Editor has decided that this paper should be retracted from the Journal. After having been in contact with the authors, they accepted the decision to retract the paper. The Editor apologizes to the readership for any inconvenience caused.

